# Wrecked regulation of intrinsically disordered proteins in diseases: pathogenicity of deregulated regulators

**DOI:** 10.3389/fmolb.2014.00006

**Published:** 2014-07-25

**Authors:** Vladimir N. Uversky

**Affiliations:** ^1^Department of Molecular Medicine and USF Health Byrd Alzheimer's Research Institute, Morsani College of Medicine, University of South FloridaTampa, FL, USA; ^2^Biology Department, Faculty of Science, King Abdulaziz UniversityJeddah, Saudi Arabia; ^3^Laboratory of New Methods in Biology, Institute for Biological Instrumentation, Russian Academy of SciencesMoscow, Russia

**Keywords:** intrinsically disordered proteins, conformational diseases, posttranslational modification, alternative splicing, transcriptional activation, expression, proteolytic degradation, trafficking

## Abstract

Biologically active proteins without stable tertiary structure are common in all known proteomes. Functions of these intrinsically disordered proteins (IDPs) are typically related to regulation, signaling, and control. Cellular levels of these important regulators are tightly regulated by a variety mechanisms ranging from firmly controlled expression to precisely targeted degradation. Functions of IDPs are controlled by binding to specific partners, alternative splicing, and posttranslational modifications among other means. In the norm, right amounts of precisely activated IDPs have to be present in right time at right places. Wrecked regulation brings havoc to the ordered world of disordered proteins, leading to protein misfolding, misidentification, and missignaling that give rise to numerous human diseases, such as cancer, cardiovascular disease, neurodegenerative diseases, and diabetes. Among factors inducing pathogenic transformations of IDPs are various cellular mechanisms, such as chromosomal translocations, damaged splicing, altered expression, frustrated posttranslational modifications, aberrant proteolytic degradation, and defective trafficking. This review presents some of the aspects of deregulated regulation of IDPs leading to human diseases.

## Introduction

For more than a 100 years, it was believed that unique protein function is defined by the unique 3-D structure of a protein, which, in its turn, is defined by a unique amino acid sequence (Fischer, 1894), and the validity of this “one sequence—one structure—one function” concept was unquestioned for a long time, especially after the crystal structures of proteins started to be solved by X-ray diffraction. However, research, especially during the past 15 years, clearly indicated that the lack of stable tertiary and/or secondary structure does not preclude proteins from being biologically active (Wright and Dyson, 1999; Uversky et al., 2000; Dunker et al., 2001; Tompa, 2002; Uversky and Dunker, 2010). In fact, many proteins with crucial biological functions exist as collapsed or extended dynamically mobile conformational ensembles and do not have unique well-defined 3D structures as a whole or in their noticeable parts (at least in *in vitro* experiments). These proteins are known as intrinsically disordered proteins (IDPs) or hybrid proteins possessing both structured domains d biologically important intrinsically disordered protein regions (IDPRs). In addition to foldable (ordered) and non-foldable (intrinsically disordered) proteins, many proteins are known to misfold. Often such misfolding is accompanied by protein aggregation causing several human diseases that originate from the deposition of protein aggregates formed from specific proteins or protein fragments, which accumulate in a variety of organs and tissues (Kelly, 1998; Bellotti et al., 1999; Dobson, 1999; Uversky et al., 1999a,b; Rochet and Lansbury, 2000; Uversky and Fink, 2004; Gasperini et al., 2012; Moreau and King, 2012; Safar, 2012; Walker and LeVine, 2012; Cuanalo-Contreras et al., 2013; Mulligan and Chakrabartty, 2013; Hipp et al., 2014). More than 20 different proteins are known so far to be involved in these diseases referred to as amyloidoses. Furthermore, many other diseases [such as cancer or cardiovascular disease (CVD)] are caused by the misfolded and therefore dysfunctional proteins (Iakoucheva et al., 2002; Cheng et al., 2006; Uversky, 2008a, 2009, 2014; Uversky et al., 2008, 2009; Uversky and Dunker, 2010). Therefore, natural proteins can be found in one of three major protein forms, functional and folded, non-functional and misfolded, and functional and intrinsically disordered.

The structural plasticity and conformational adaptability of IDPs/IDPRs, their ability to react easily and quickly in response to changes in their environment, and their binding promiscuity and unique capability to fold differently while interacting with different binding partners (Dyson and Wright, 2005; Oldfield et al., 2008) define a wide set of functional advantages of IDPs/IDPRs over the ordered proteins (Uversky and Dunker, 2010; Cozzetto and Jones, 2013; Ferreon et al., 2013). These factors determine the abundant involvement of IDPs/IDPRs in various signaling, regulation, and recognition processes. They also allow these flexible proteins to play diverse roles in modulation and control of functions of their binding partners and in promotion of the assembly of supra-molecular complexes. In fact, IDPs are promiscuous binders and can form highly stable complexes, or be involved in signaling interactions where they undergo constant “bound-unbound” transitions, thus acting as dynamic and sensitive “on-off” switches. The ability of these proteins to return to the highly flexible conformations after the completion of a particular function, and their predisposition to adopt different conformations depending on their environment, are unique physiological properties of IDPs which define the ability of these proteins to exert different functions in different cellular contexts according to a specific conformational state (Uversky and Dunker, 2010).

Furthermore, biological activities of IDPs/IDPRs are known to be precisely and tightly controlled and regulated by extensive posttranslational modifications (PTMs), such as phosphorylation, acetylation, glycosylation, etc. (Collins et al., 2008; Uversky and Dunker, 2010; Kurotani et al., 2014; Pejaver et al., 2014), and by alternative splicing (AS) (Romero et al., 2006; Buljan et al., 2012, 2013). The ability of AS to generate extended sets of protein isoforms with highly diverse regulatory elements (Romero et al., 2006) is determined by the mosaic structure of IDPs/IDPRs that are known to contain multiple relatively short, functional elements, which, being spread within the amino acid sequences, define the multifunctionality of these proteins (Uversky, 2013). Clearly, AS-driven removal of pieces of the IDP/IDPRs sequence containing different functional elements could dramatically reshuffle such multifunctionality (Buljan et al., 2012, 2013; Colak et al., 2013).

Careful examination of different proteomes and various large protein datasets (e.g., UniProt database, http://web.expasy.org/docs/swiss-prot_guideline.html) by various disorder predictors revealed that IDPs are not some obscure and rare exceptions from the general “one sequence—one unique structure—one unique function” paradigm (Wright and Dyson, 1999; Uversky et al., 2000; Dunker et al., 2001; Tompa, 2002), but, in fact, are highly abundant in nature (Dunker et al., 2000; Ward et al., 2004; Tompa et al., 2006; Krasowski et al., 2008; Shimizu and Toh, 2009; Tokuriki et al., 2009; Pentony and Jones, 2010; Tompa and Kalmar, 2010; Uversky, 2010a; Xue et al., 2010a, 2012a; Dyson, 2011; Schad et al., 2011; Hegyi and Tompa, 2012; Korneta and Bujnicki, 2012; Midic and Obradovic, 2012; Pancsa and Tompa, 2012; Di Domenico et al., 2013; Kahali and Ghosh, 2013). These natural abundance of IDPs and IDPRs are now documented in multiple entries populating various disorder-related databases, such as DisProt (Vucetic et al., 2005; Sickmeier et al., 2007), D^2^P^2^ (Oates et al., 2013), Ideal (Fukuchi et al., 2012, 2014), MobiDB (Di Domenico et al., 2012), ComSin (Lobanov et al., 2010), and pE-DB (Varadi et al., 2014). The overall amount of IDPs and IDPRs in various proteomes increases with the increase in the organism's complexity, with over half of all eukaryotic proteins predicted to contain long IDPRs (Dunker et al., 2000; Ward et al., 2004; Oldfield et al., 2005; Uversky, 2010a; Xue et al., 2012a). It is believed that the potential explanation for this trend has its roots in a change in the cellular requirements for certain protein functions, particularly for proteins involved in cellular signaling. In agreement with this hypothesis, a computational analysis revealed that the majority of known eukaryotic signaling proteins contain significant regions of disorder (Dunker et al., 2002; Iakoucheva et al., 2002).

Many individual IDPs and hybrid proteins (e.g., α-synuclein, p53, PTEN, etc.) are known to interact with large number of unrelated partners, thereby serving as hubs in cellular protein-protein interaction networks (Dunker et al., 2005; Uversky et al., 2005). Also, the binding regions of partner proteins interacting with structured hubs, such as 14-3-3 and calmodulin, are often intrinsically disordered (Bustos and Iglesias, 2006; Radivojac et al., 2006). Therefore, there are two general mechanisms by which intrinsic disorder is exploited in protein-protein interaction networks, where one IDP/IDPR binds to many partners and many IDPs/IDPRs interact with one partner (Dunker et al., 2005; Uversky et al., 2005). Subsequent comprehensive bioinformatics studies supported the hypothesis that hub proteins commonly use disordered regions to bind to multiple partners (Dosztanyi et al., 2006; Ekman et al., 2006; Haynes et al., 2006; Patil and Nakamura, 2006; Singh and Dash, 2007; Singh et al., 2007).

## IDPs in human diseases

### A general overview of the IDP involvement in pathogenesis of various maladies

Since IDPs/IDPRs are very common in all proteomes studied to date, possess numerous crucial functions, are promiscuous binders, are abundantly involved in signaling, control, and regulation of important biological processes, where it is crucial for a given protein to be available in appropriate amounts and not to be present longer than needed, IDPs and hybrid proteins have to be tightly regulated and controlled themselves. To check this hypothesis, careful analysis of the IDP regulation inside the cell at different stages of protein synthesis and degradation was recently conducted using the corresponding data available for the *Saccharomyces cerevisiae, Schizosaccharomyces pombe*, and *Homo sapiens* proteomes (Gsponer et al., 2008). This analysis revealed that IDPs were less abundant than ordered proteins in these tree proteomes because of the increased decay rates of mRNAs encoding IDPs, lower rates of IDP protein synthesis, and shorter half-lives of IDPs (Gsponer et al., 2008). Also, the majority of IDP-targeting kinases were either regulated in a cell-cycle dependent manner, or were activated upon exposure to specific stimuli or stress (Gsponer et al., 2008), thereby adding another level of the IDP control. It is important to remember though that although the abundance of many IDPs is tightly regulated, some disordered, and hybrid proteins are present in cells in large amounts [for example, α-synuclein constitutes ~1% of the total soluble protein in the brain (Iwai et al., 1995)] or/and for long periods of time due to either specific PTMs, or via interactions with other factors, or due to the localization is specific compartments (Uversky and Dunker, 2010). These events could promote changes in cellular localization of IDPs or protect them from degradation (Dunker et al., 2001; Iakoucheva et al., 2004; Tompa, 2005; Grimmler et al., 2007). Therefore, accumulated data clearly show that the chaos seemingly associated with highly flexible and promiscuous IDPs/IDPRs is under tight control (Uversky and Dunker, 2008).

In addition to mentioned bioinformatics analyses numerous experimental studies not only emphasize the important roles of disordered regulators in signaling (Mitrea and Kriwacki, 2013; Follis et al., 2014), regulation (Galea et al., 2008a; Wang et al., 2011; Follis et al., 2012; Mitrea et al., 2012; Ou et al., 2012; Yoon et al., 2012; Frye et al., 2013; Moldoveanu et al., 2013), cell protection (Mei et al., 2014), protein protection (De Jonge et al., 2009; Chakrabortee et al., 2012), and cellular homeostasis (Norholm et al., 2011; Follis et al., 2013), but also show that IDPs/IDPRs are concisely controlled by themselves via multiple mechanisms, such as interaction with chaperones (Rudiger et al., 2002; Martinez-Yamout et al., 2006; Rodriguez et al., 2008; Didenko et al., 2012; Karagoz et al., 2014) or nanny proteins (Tsvetkov et al., 2009a), partner binding (Demarest et al., 2002; Chipuk et al., 2008; Ebert et al., 2008; Ferreon et al., 2009a,b; Wojciak et al., 2009; Lee et al., 2010), various PTMs (Kostic et al., 2006; Grimmler et al., 2007; Zhan et al., 2007; Galea et al., 2008b; Mitrea et al., 2014), and regulated degradation (Asher et al., 2005; Tsvetkov et al., 2008, 2009b, 2012; Suskiewicz et al., 2011; Wiggins et al., 2011).

Obviously, when tightly controlled process is suddenly coming out of control, consequences could be disastrous. In agreement with this statement, numerous cases are known where the malfunction of a protein (which could be ordered or intrinsically disordered) is associated with the development of particular pathological conditions, and a broad range of human diseases is linked to the failure of a specific peptide or protein to adopt its functional conformational state. Each of these conformational diseases originates from the dysfunction of a particular protein. The failure of such a protein to adopt, possess, or keep functional state is commonly associated with protein misfolding, loss of normal function, gain of toxic function, and/or protein aggregation (Uversky et al., 2009; Uversky, 2010b). Some disease-related proteins have an intrinsic propensity to form pathologic conformation(s). For other proteins, interactions or impaired interactions with chaperones, intracellular or extracellular matrices, other proteins, small molecules, and other endogenous factors can induce conformational changes and increase propensity to misfold. Often, misfolding, and dysfunction originate from point mutation(s) or result from a protein exposure to internal or external toxins. Furthermore, such conformational diseases can also be caused by impaired PTMs, such as phosphorylation, advanced glycation, deamidation, racemization, etc., an increased probability of degradation, impaired trafficking, loss of binding partners, or oxidative damage. All these factors can act independently, additively, or synergistically (Uversky, 2012).

It is recognized now that IDPs and hybrid proteins with long IDPRs are commonly involved in human diseases. For example, some well-known cancer-related proteins with experimentally confirmed IDPRs include p53 (Wells et al., 2008), BRCA1 (Mark et al., 2005), EWS (Ng et al., 2007), HPV protein (Uversky et al., 2006), PTEN (Malaney et al., 2013a), axin (AXis Inhibition) (Noutsou et al., 2011; Xue et al., 2012b, 2013), adenomatous polyposis coli (APC) protein (Xue et al., 2012b; Minde et al., 2013), apoptosis-stimulating proteins of p53 (ASPPs) (Rotem et al., 2007; Ahn et al., 2009), BH3-only proteins (Kvansakul and Hinds, 2014), sirtuins (McBurney et al., 2013; Sharma et al., 2013), CBP/?p300 [CREB-binding protein (CBP) and its paralog, E1A-binding protein p300] (Wojciak et al., 2009), cell cycle regulatory proteins p21 and p27 (Mitrea et al., 2012), and many others. Recent computational analysis revealed that a majority of the cancer/testis antigens (CTAs) are typical IDPs (Rajagopalan et al., 2011). CTAs constitute a heterogeneous protein group, members of which are typically expressed in the normal testis and aberrantly expressed in several types of cancer (Rajagopalan et al., 2011). Among the neurodegeneration-related proteins is such well-characterized IDPs as a protein-chameleon α-synuclein that can adopt a variety of different conformations, starting from random coil and ending with a more compact molten globular state, or even with poly-(L-proline) II-like conformations, depending on the cellular environment (Uversky, 2003), and aggregates of the α-synuclein are accumulated in Parkinson's disease, dementia with Lewy bodies, Alzheimer's disease (AD), Down's syndrome, and several other synucleinopathies (Uversky, 2007, 2008b; Uversky and Eliezer, 2009; Breydo et al., 2012; Alderson and Markley, 2013). Other IDPs implicated in neurodegenerative diseases include amyloid β and tau proteins (AD), prions (Creutzfeldt-Jakob disease, scrapie, bovine spongiform encephalopathy), and ataxin (spinocerebellar ataxia) (Uversky, 2009, 2014). Other human diseases with well-established pathogenic IDPs are CVDs (hirudin and thrombin) (Cheng et al., 2006), type II diabetes (amylin) (Uversky et al., 2008), AIDS (HIV Rev protein) (Casu et al., 2013), and cystic fibrosis (CFTR) (Baker et al., 2007).

### Computational approaches for estimating the IDP abundance in different diseases

The fact that IDPs and hybrid proteins with long IDPRs are commonly associated with the pathogenesis of various human disorders gave rise to the “disorder in disorders” or D^2^ concept (Uversky et al., 2008). Here, the dysfunction, misidentification, misregulation, misfolding, and missignaling of causative IDPs/IDPRs are all can be considered as causative factors of the conformational diseases (Uversky et al., 2008, 2009, 2014; Uversky, 2009, 2010b, 2012; Midic et al., 2009a,b). Although the fact of the involvement of individual IDPs and hybrid proteins with long IDPRs in the pathogenesis of some human maladies is well-established, the application of specially designed computational and bioinformatics protocols opens a unique opportunity for the accurate evaluation of the abundance of IDPs in various pathological conditions.

The first of these computational approaches is based on the assembly of specific datasets of proteins associated with a given disease and the computational analysis of these datasets using a number of disorder predictors (Iakoucheva et al., 2002; Cheng et al., 2006; Uversky et al., 2006; Mohan et al., 2008; Uversky, 2008a, 2009). This approach represents an extension of the analysis of individual proteins to a set of independent proteins. Such analysis revealed that 79% of cancer-associated and 66% of cell-signaling proteins contain predicted regions of disorder of 30 residues or longer (Iakoucheva et al., 2002). Similar analyses revealed that the percentage of proteins with 30 or more consecutive disordered residues was 61% for proteins associated with CVD (Cheng et al., 2006). Many CVD-related proteins were predicted to be entirely disordered, with 101 proteins from the CVD dataset predicted to have a total of almost 200 specific disorder-based binding motifs (thus about 2 binding sites per protein) (Cheng et al., 2006). Finally, the dataset analysis revealed that intrinsic disorder is commonly found in neurodegenerative diseases and diabetes (Uversky et al., 2008, 2009).

In a second approach, the abundance of intrinsic disorder was analyzed in the human diseasome (Midic et al., 2009a), which is a complex network that systematically links the human disease phenome with the human disease genome (Goh et al., 2007). These analyses showed that many human genetic diseases are caused by alterations of IDPs, that different disease classes vary in the disorder contents of their associated proteins, and that many IDPs involved in some diseases are enriched in disorder-based protein interaction sites (Midic et al., 2009a,b).

Finally, a third approach is based on the evaluation of the association between the level of intrinsic disorder and a particular protein function (including the disease-specific functional keywords) in a set of proteins known to carry out this function (Vucetic et al., 2007; Xie et al., 2007a,b). In this approach, it is hypothesized that if intrinsic disorder is important for a function described by a given keyword, then, a greater level of predicted disorder would be found in a protein associated with this keyword than the level of disorder predicted in a protein randomly chosen from the Swiss-Prot (Vucetic et al., 2007; Xie et al., 2007a,b). To test this hypothesis, functional keywords associated with at least 20 Swiss-Prot proteins were found and corresponding keyword-associated datasets of proteins were assembled. For each keyword-associated set, 1000 length-matching and number-matching sets of random proteins were drawn from Swiss Prot. Order-disorder predictions were carried out for the keyword-associated sets and for the matching random sets. If a function described by a given keyword were carried out by a long region of disordered protein, one would expect the keyword-associated set to have a greater amount of predicted disorder compared to the matching random sets. The keyword-associated set would be expected to have less prediction of disorder compared to the random sets if the keyword-associated function were carried out by structured protein. Given the predictions for the function-associated and matching random sets, it is possible to calculate the *p*-values, where a *p*-value > 0.95 suggests a disorder-associated function, a *p*-value < 0.05 suggests an order-associated function, and intermediate *p*-values are ambiguous (Vucetic et al., 2007; Xie et al., 2007a,b). This analysis revealed that out of 710 Swiss-Prot keywords, 310 functional keywords were associated with ordered proteins, 238 functional keywords were attributed to disordered proteins, and the remainder 162 keywords yield ambiguity in the likely function-structure associations (Vucetic et al., 2007; Xie et al., 2007a,b). Furthermore, many diseases were found to be strongly correlated with proteins predicted to be disordered (Vucetic et al., 2007; Xie et al., 2007a,b). Contrary to this, almost no disease-associated proteins were found to be ordered (Xie et al., 2007a).

## Wrecked regulation of IDPs and disease

**Figure 1:**
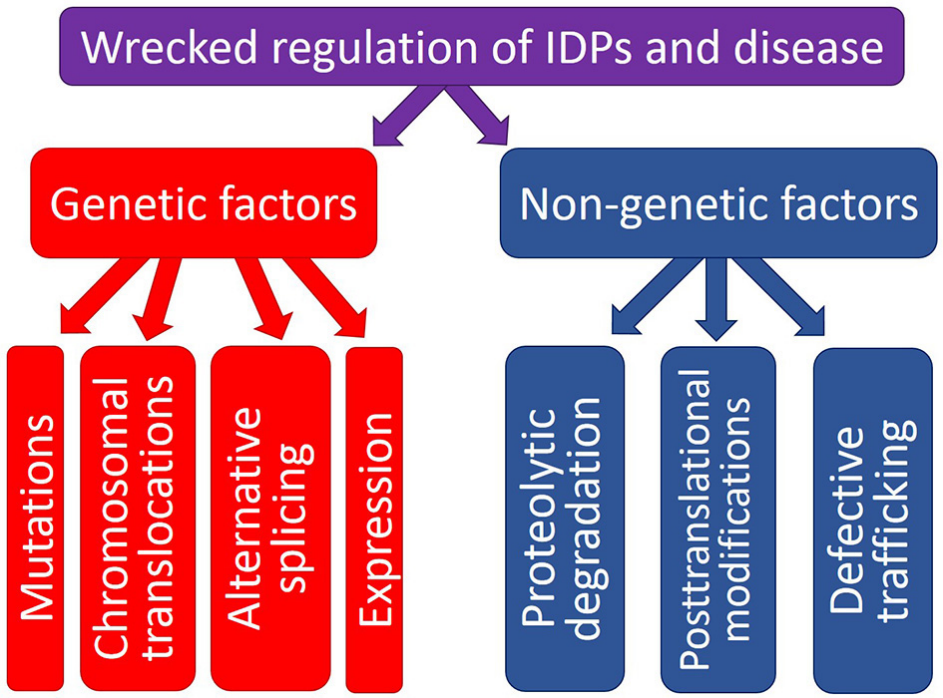
**Schematic representation of different ways by which aberrant regulation at the genetic level or various posttranslational (non-genetic) mechanisms can cause pathogenic transformation in an IDP/IDPR**.

The physiological protein function and the ability to be converted from a normal protein to a pathological form depend on multiple factors which can be grouped into two major classes, genetic and non-genetic. Genetic factors include pathological mutations, aberrant splicing, chromosomal translocation, alternative transcription, and altered AS. Non-genetic factors are related to the peculiarities and levels of protein expression, protein availability, regulation, interaction patterns, cleavage propensity, and PTMs. Some illustrative examples of these transforming factors leading to the appearance of pathological proteins are given below. Since the association between the pathological mutations and IDPs/IDPRs was a subject of several research papers and reviews, this mechanism of affecting the physiological protein function will not be discussed in this review, and the interested readers are encouraged to look for the corresponding information in the original works (e.g., Joerger and Fersht, 2007, 2008; Uversky, 2008b; Midic et al., 2009a,b; Vacic and Iakoucheva, 2012; Vacic et al., 2012; Yates and Sternberg, 2013). Figure 1 is a scheme illustrating different ways by which aberrant regulation of IDPs/IDPRs at different levels can result in the development of pathological conditions.

### Genetic factors: chromosomal translocations

**Figure 2:**
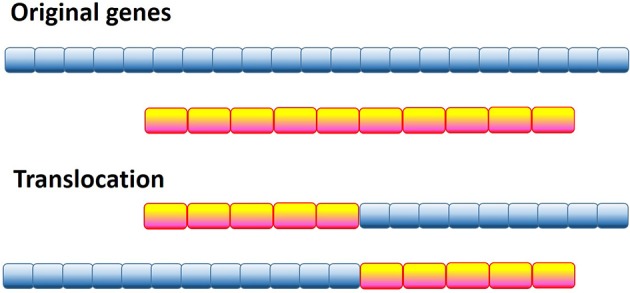
**Schematic representation of the genetic translocation mechanism, where instead of two normal functional proteins a hybrid protein is produced with aberrant biological activity**.

One of the most radical and obvious ways to generate a pathological protein is chromosomal translocation, which generates chimeric proteins by fusing segments of two otherwise separated genes (see Figure 2). Several forms of cancer, such as acute myelogenous leukemia (AML), acute lymphoblastic leukemia (ALL), chronic myelogenous leukemia (CML), chronic myelomonocytic leukemia (CMML), primary myelofibrosis (PMF), anaplastic large cell lymphoma, non-Hodgkin's lymphoma, Ewing's sarcoma (EWS), colorectal cancer (CRC), non-small cell lung cancer, lung adenocarcinoma, and sporadic and radiation-associated papillary thyroid carcinomas are caused by chromosomal translocation. Computational analysis of the 406 translocation-related human proteins revealed that these oncoproteins are significantly enriched in intrinsic disorder, with the translocation breakpoints being mostly located outside the functional domains (Hegyi et al., 2009). Furthermore, the vicinities of the breakpoint were shown to be even more disordered than the rest of these already highly disordered fusion proteins. These observations suggest that high levels of intrinsic disorder represents an important factor that helps fusion proteins to escape detection by cellular surveillance mechanisms that eliminate misfolded proteins and to live long enough to manifest their altered function(s) (Hegyi et al., 2009).

The authors found that these translocation-generated fusions enable the long-range structural communication of remote binding and/or catalytic domains in the chimeric proteins and thereby define the acquired oncogenic functions. One of the illustrative examples of such acquired oncogenicity is the acquired intramolecular phosphorylation of the BCR-ABL fusion protein related to CML and ALL. Here, chromosomal translocation results in fusion of a Tyr-kinase phosphorylation motif in BCR with the Tyr-kinase domain within ABL, with disorder of the intervening region enabling intramolecular phosphorylation (Hegyi et al., 2009). Another recent example in this category is the BCR-RET chimeric protein originated from the fusion of the catalytic domain of the tyrosine kinase (TK) receptor RET (REarranged during Transfection) with BCR (Ballerini et al., 2012).

The second mechanism of generation of oncoproteins by chromosomal translocation is related to fusion of the dimerization domain with the kinase domain, generating a dimeric hybrid protein. Monomers comprising this protein are engaged in multiple mutual intermolecular phosphorylation reactions that promote auto-activation and generate novel binding sites for signaling proteins. Examples of this mechanism include various chimeric proteins, such as constitutively activated TEL-JAK2 fusion (ETS translocation variant 6 – Janus tyrosine kinase 2 fusion) with kinase activity in human leukemia (Lacronique et al., 1997); TFG-ALK (TRK-fused gene - anaplastic lymphoma kinase fusion) related to anaplastic large cell lymphomas (Roccato et al., 2003); NPM-ALK (nucleolar phosphoprotein nucleophosmin – anaplastic lymphoma kinase fusion), the chimeric protein that is created by translocation in non-Hodgkin's lymphoma and that requires the activation of its ALK kinase function as a result of oligomerization mediated by the NPM segment (Bischof et al., 1997); EML4-ALK (echinoderm microtubule-associated protein-like 4 − anaplastic lymphoma kinase fusion) found in non-small lung carcinoma (Soda et al., 2007); *TPM3-ALK* (Lamant et al., 1999), *TFG-ALK* (Hernandez et al., 1999), and *ATIC-ALK* (Trinei et al., 2000) fusions in anaplastic large cell lymphoma (ALCL) (Marino-Enriquez and Dal Cin, 2013). Other ALK fusion partners include tropomyosin 4 (TPM4); clathrin heavy chain (CLTC); moesin (MSN); the cysteinyl-tRNA synthetase (CARS); RNF213, a ring finger protein; RAN binding protein 2 (RANBP2); myosin II heavy chain type A (MYH9); vinculin (VCL); dynactin (DCTN1); liprin beta 1 (PPFIBP1); KIF5B, a kinasin family member; KLC1, kinesin light chain 1; and sequestosome 1 (SQSTM1) (Marino-Enriquez and Dal Cin, 2013). In the vast majority of the ALK-based fusions, the chimeric proteins include all 563 cytoplasmic amino acids from ALK (residues 1058–1620), which is the constitutively active C-terminal tyrosine kinase domain (Marino-Enriquez and Dal Cin, 2013), whereas the ALK fusion partner determines the subcellular localization of the fusion oncoprotein via its peptide localization signals and its homotypic or heterotypic oligomerization domains, which in turn will condition the protein-protein interactions and modulate the oncogenic signaling and molecular consequences of the ALK oncoprotein action (Marino-Enriquez and Dal Cin, 2013). Another highly promiscuous chromosomal translocator is RET, the catalytic domain of which, in addition to the aforementioned BCR-RET fusion, is fused with the heterologous oligomerization domains encoded by different genes, such as *H4* (for *RET-PTC1*), *RI*α (*RET*-*PTC2*), *ELE1* (*RET*-*PTC3* and *RET*-*PTC4*), *RFG5* (*RET*-*PTC5*), *hTIF* (*RET*-*PTC6*), *RFG7* (*RET*-*PTC7*), *kinectin* (*RET*-*PTC8*), *RFG9* (*RET*-*PTC9*), and *ELKS* (*ELKS*-*RET*) (Takahashi, 2001), with *KIF5B* (*KIF5B-RET*), or fusion with the fibroblast growth factor receptor 1 (FGFR1) oncogene partner (FGFR1OP-RET) (Bossi et al., 2014). In the case of FGFR1OP-RET fusion, the chimeric protein found in papillary thyroid carcinomas and other myeloproliferative disorders, such as CML, chronic eosinophilic leukemia, chronic neutrophilic leukemia, polycytemia vera, PMF, essential thrombocytosis, myelodysplastic syndromes (MDS), and CMML was shown to display constitutive tyrosine kinase and transforming activity (Bossi et al., 2014).

Chromosomal translocation can affect transcription factors, as illustrated by the EWS-ATF or EWS-Fli1 hybrids, where the DNA-binding elements of transcription factors ATF1 or Fli1 are fused to the disordered transactivation domain of the EWS oncogene to generate an aberrant transcription factor related to Ewing sarcoma (Ng et al., 2007). Another very interesting example of the transcription factor whose function is affected by chromosomal translocation is the ETV6 protein containing two major domains, the HLH (helix-loop-helix, residues 40–124) domain and the ETS domain (residues 340–420) connected by the internal domain (residues 125–339) (De Braekeleer et al., 2012), whose gene spans a region of less than 250 kb at band 12p13.1 and consists of 8 exons (Baens et al., 1996). The importance of this protein is determined by the fact that the *ETV6* chromosomal translocations are among the most commonly observed chromosomal abnormalities in human leukemia and myelodysplastic syndrome (Baens et al., 1996), where there are 48 chromosomal bands involved in *ETV6* translocations, insertions, or inversions, with at least 28 translocations being characterized at the molecular level and 30 *ETV6* partner genes being identified (De Braekeleer et al., 2012). In fact, the *ETV6* gene is known to be fused with a wide array of genes encoding proteins with different functionalities, such as receptor tyrosine kinase genes (e.g., *ETV6-PDGFRB, ETV6-PDGFRA, ETV6-NTRK3*, and *ETV6-FLT3*), non-receptor tyrosine kinase genes (such as *ETV6-ABL1, ETV6-ABL2, ETV6-JAK2, ETV6-FGFR3, ETV6-SYK, ETV6-FRK*, and *ETV6-LYN*), transcription factor genes (such as *ETV6-RUNX1, MN1-ETV6, ETV6-ARNT, ETV6-PER1*, and *ETV6-EVI1*), homeobox genes (such as *ETV6-CDX2, PAX5-ETV6*, and *MNX1-ETV6*) and many other genes (e.g., *CHIC2-ETV6, ETV6-MDS2, TTL-ETV6, ETV6-STL, ETV6-PTPRR, ETV6-NCOA2, ETV6-BAZ2A, ETV6-GOT1, ETV6-FCHO2, ETV6-IGH*, and *ETV6-ACSL6*) (De Braekeleer et al., 2012). It is important to emphasize here that the mentioned fusions do not include the full-length ETV6 protein. Furthermore, even fusions of the *ETV6* gene with the same target gene not always will have the same parts of the ETV6 protein. For example, upon fusion to the *PDGFRB* gene encoding a cell surface tyrosine kinase receptor for members of the platelet-derived growth factor family, the resulting *ETV6-PDGFRB* fusion gene in myeloproliferative disorders will have exon 4 of the *ETV6* gene fused in-frame to exon 11 of the *PDGFRB* gene, with the resulting chimeric protein containing the HLH domain of ETV6 [ETS translocation variant 6 (also known as TEL), N-terminal part] and the tyrosine kinase domain of PDGFRB (platelet-derived growth factor receptor beta, C-terminal part), whereas in chronic idiopathic myelofibrosis (CIMF) the fusion of the same two genes will produce in-frame fusion between full-length *ETV6* exon 7, 34 bp derived from *ETV6* intron 7 and a truncated *PDGFRB* exon 12, giving rise to the chimeric protein retaining the internal domain of ETV6 which has the ability to bind to corepressors and induce the transcription-repressive activity of ETV6 (De Braekeleer et al., 2012).

**Figure 3:**
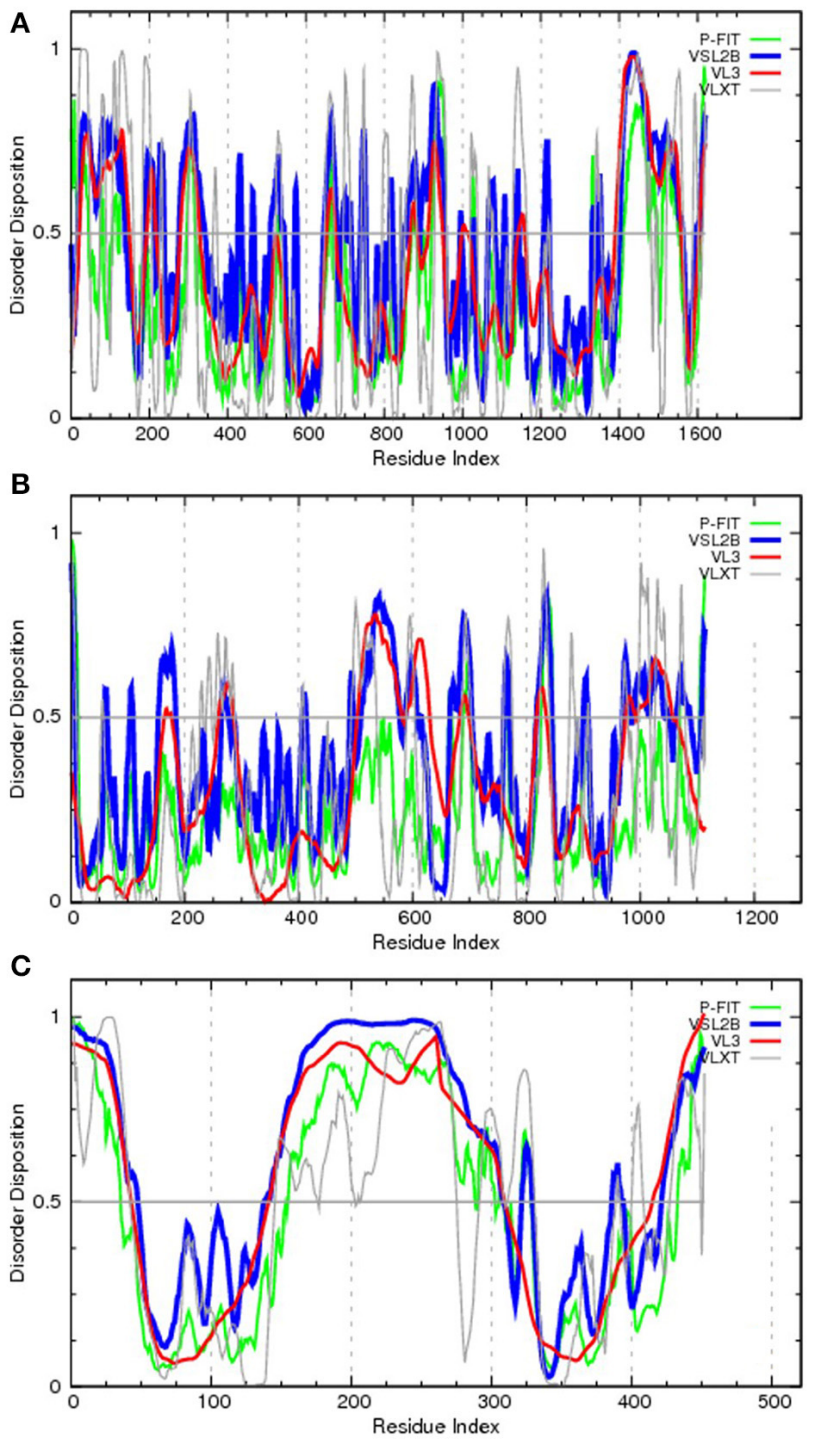
**Evaluating the disorder propensities of proteins commonly affected by chromosomal translocation: ALK (A), RET (B), and ETV6 (C)**. The disorder propensities are evaluated by the members of the PONDR family of disorder predictors. Here, scores above 0.5 correspond to disordered residues/regions. PONDR^®^ VSL2B is one of the most accurate stand-alone disorder predictors (Obradovic et al., 2005), PONDR^®^ VL3 possesses high accuracy in finding long IDPRs (Obradovic et al., 2003), PONDR^®^ VLXT is not the most accurate predictor but has high sensitivity to local sequence peculiarities which are often associated with disorder-based interaction sites (Dunker et al., 2001), whereas PONDR-FIT represents a metapredictor which, being moderately more accurate than each of the component predictors, is one of the most accurate disorder predictors (Xue et al., 2010b).

To bring the aforementioned data on the chromosomal translocation to the frames of this review, Figure 3 represents disorder propensities of some of the most promiscuous chromosomal translocators, ALK, RET, and ETV6. Figure 3A shows that the C-terminal domain of ALK, which is included in the vast majority of ALK-based chimeric proteins, is predicted to have highly disordered N-terminal and C-terminal tails. Figure 3B illustrates that the same statement is also applicable to RET, catalytic domain of which (residues 708–114) is fused to many target proteins and is predicted to be surrounded by long IDPRs. Finally, Figure 3C shows that the transcription factor ETV6 is predicted to have two ordered domains (residues 43–145 and 310–420), which coincide with known functional domains of this protein, PNT domain (residues 40–124) and DNA-binding ETS domain (residues 339–420).

### Genetic factors: aberrant RNA splicing

#### Intrinsic disorder and alternative splicing

Alternative splicing (AS) of pre-mRNAs, which generates two or more protein isoforms from a single gene, is believed to be responsible for tissue specificity of many of the abundant proteins. Estimates indicate that between 35 and 60% of human genes yield protein isoforms by means of AS mRNA (Stamm et al., 2005). Regions of mRNA affected by AS were shown to correspond to protein regions enriched in intrinsic disorder (Romero et al., 2006). The finding that the alternatively spliced regions of mRNA encode IDPRs with greater frequencies than structured regions suggests a link between AS and signaling by IDPRs. This connection constitutes a plausible mechanism that could underlie and support cell differentiation, which ultimately gave rise to the multicellular eukaryotic organisms (Romero et al., 2006). Furthermore, associating AS with protein disorder enables time- and tissue-specific modulations of protein functions. Since disorder is frequently utilized in protein binding regions, having AS of pre-mRNA coupled to regions of protein disorder can lead to tissue-specific signaling and regulatory diversity (Romero et al., 2006; Weatheritt and Gibson, 2012). In agreement with this hypothesis, recent bioinformatics analysis clearly showed that tissue-specific AS of IDPRs with embedded binding motifs is responsible for rewiring of protein interaction networks and signaling pathways (Buljan et al., 2012, 2013). Also, in the human proteome, two types of conserved IDPRs, conserved disorder (i.e., disordered regions where the amino acid sequence is well conserved) and flexible disorder (i.e., disordered regions where the amino acid sequence has diverged) were shown to be highly abundant in proteins with regulatory functions and were highly enriched in regions of proteins that undergo tissue-specific AS (Colak et al., 2013).

#### Altered alternative splicing and diseases

**Figure 4:**
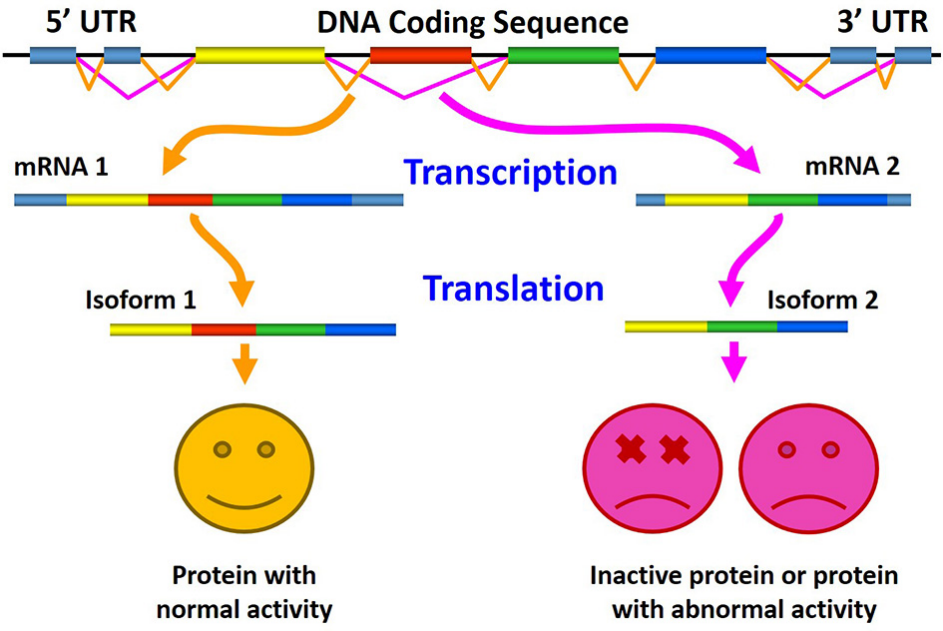
**Schematic representation of the role of aberrant alternative splicing in production of pathogenic proteins**. Here, normal splicing results in the production of biologically active protein **(left)**, whereas altered alternative splicing generates either inactive protein or a splice variant with an abnormal (pathological) activity **(right)**.

The role of aberrant AS in generating pathogenic proteins is shown in Figure 4, where the normal splicing results in the production of normally active protein (left), and where altered AS generates either inactive protein or a splice variant with pathological or abnormal activity (right).

***Aberrant alternative splicing in cancer***. Although the flexibility of AS constitutes an evolutionary advantage for higher eukaryotes, it also represents a risk. In fact, strong evidence indicates that defective AS regulation correlates with the onset and progression of human cancers (Lee et al., 2006; David and Manley, 2010; Pal et al., 2012; Chen and Miller, 2013; Shkreta et al., 2013; Biamonti et al., 2014; Chen and Weiss, 2014), and many cancer-associated genes are regulated through AS suggesting a significant role of this post-transcriptional regulatory mechanism in the production of oncogenes and tumor suppressors (Bonomi et al., 2013). Therefore, not surprisingly, aberrant AS is considered now as an important hallmark of cancer (Ladomery, 2013). The phenomenon of cancer-associated (or cancer-promoting) aberrant splicing is widespread. For example, such aberrant AS events have been found in tumor suppressor LKB1, reduced levels of which are found in patients with Peutz-Jeghers Syndrome (PJS), an autosomal dominant disorder associated with gastrointestinal polyposis and an increased cancer risk, oncogene KIT associated with gastrointestinal stromal tumors, cell-cell adhesion protein CDH17 overexpressed in hepatocellular carcinomas (HCCs) and in gastric and pancreatic cancer, and a Kruppel-like Zn-finger transcription factor KLF6 that functions as a tumor suppressor (Srebrow and Kornblihtt, 2006), as well as in genes implicated in tumor progression (for example, CD44, MDM2, and FHIT) and in susceptibility to cancer [for example, APC (Adenomatous polyposis coli protein)] (Kalnina et al., 2005).

Also, ~29% of genome-wide expressed genes were shown to be differentially and recurrently spliced in AML patients compared to healthy individuals (Adamia et al., 2014). Among these differentially spliced genes were genes encoding several oncogenes, tumor suppressor proteins, splicing factors and heterogeneous nuclear ribonucleoproteins, proteins involved in apoptosis, cell proliferation, and spliceosome assembly (Adamia et al., 2014). Many of these targets of the aberrant splicing in AML, are proteins with known enrichment in intrinsic disorder, such as proteins related to apoptosis (Peng et al., 2013) as well as proteins involved in spliceosome assembly (Korneta and Bujnicki, 2012; Coelho Ribeiro Mde et al., 2013). Some of the crucial proteins affected by AS in prostate cancer [e.g., the androgen receptor (AR), zinc finger transcription factor Kruppel-like factor 6, Bcl-x, and cyclin D1] (Sette, 2013) are known to contain IDPRs (McEwan, 2012; Peng et al., 2013).

Three major players in the prevention of breast cancer are p53, BRCA1, and PTEN. Mutations in the p53, BRCA1, and PTEN genes account for about 10% of familial breast and ovarian cancer cases overall (Muggia et al., 2011; Okumura et al., 2011). Furthermore, genes of these proteins undergo extensive AS events and some splicing variants of these tumor suppressor genes are associated with cancer (Okumura et al., 2011). These three proteins are known to possess functionally important IDRPs. Since they are among the well-characterized protein with disorder, they are considered in more details below.

Although p53 is best known for its role as a tumor suppressor and guardian of the genome (Lane, 1992), besides induction of cell-cycle arrest, apoptosis, or DNA repair, it is also involved in many other cellular processes, including senescence and differentiation (Vousden and Prives, 2009). p53 gene is expressed as multiple isoforms (with sometimes antagonistic functions) due to AS, alternative promoter usage and alternative initiation of translation (Bourdon, 2007). The human p53 gene transcribes multiple splice variants, which are differentially expressed in human breast tumors compared with normal breast tissue (Okumura et al., 2011) and also are differentially expressed from tumor to tumor (Bourdon et al., 2005). There are four domains in p53: the unfolded N-terminal translational activation domain, the structured central DNA binding domain, and the unstructured C-terminal tetramerization and regulatory domain (Oldfield et al., 2008; Uversky and Dunker, 2010). At the transactivation region, p53 interacts with TFIID, TFIIH, Mdm2, RPA, CBP/p300, and CSN5/Jab1 among many other proteins (Anderson and Appella, 2004). At the C-terminal domain, it interacts with GSK3β, PARP-1, TAF1, TRRAP, hGcn5, TAF, 14-3-3, S100B(ββ) and many other proteins (Anderson and Appella, 2004). p53 serves as a unique example illustrating how intrinsic disorder brings about binding promiscuity, since the same short segment near the p53 C-terminus, being disordered in its unbound form, was shown to bind to four different partners and to adopt different bound conformations. In fact, this disordered segment adopts an α-helix, a β-strand, and two irregular structures with very different morphology upon binding to its four different partners (Oldfield et al., 2008; Uversky and Dunker, 2010).

Another important player in the controlling the breast cancer development is BRCA1 (breast cancer type 1 susceptibility protein), which is the 1863 amino acids protein with an amino terminal zinc ring finger motif, two nuclear localization signals, and two C-terminally located BRCT domains. Comprehensive structural and functional analysis revealed that the long central region of BRCA1 (residues 170–1645) represents an intrinsically disordered scaffold for multiple protein-protein and protein-DNA interactions (Mark et al., 2005). Dysregulation of AS of BRCA1 has also been indicated to be involved in the formation of the breast and ovary tumors (Orban and Olah, 2001; Okumura et al., 2011).

Finally, a few words about PTEN (phosphatase and tensin homolog, deleted on chromosome TEN), which is a tumor suppressor on 10q23.3 mutated in many types of cancers (Blumenthal and Dennis, 2008; Carracedo et al., 2011; Hollander et al., 2011). In fact, PTEN is the second most frequently mutated tumor suppressor gene after p53 (Uversky et al., 2014). Also, differential expression of PTEN and its splice variants can play a role in the pathogenesis of sporadic breast cancers (Okumura et al., 2011). For example, the PTEN splice variants retained intron 3 regions (SV3) and intron 5 regions (SV5) have been found in breast cancers (Okumura et al., 2011). Structurally, PTEN consists of the intrinsically disordered N-terminal phosphatidyl inositol (Dunker et al., 2001; Tompa, 2002)-bisphosphate (PIP2) binding module (PBM), which allows PTEN to anchor to the membrane (its site of action) via PIP2 molecules; the dual-specificity lipid and protein phosphatase domain (PD), which contains a conserved catalytic motif HCKAGKGR; the C2 domain, consisting of positively charged residues that help PTEN to associate with phosphatidylserine residues in the plasma membrane; and the C-terminal intrinsically disordered region (C-tail), which regulates membrane association and stability through several phosphorylation modifications (Malaney et al., 2013a). A longer variant of PTEN (PTEN-long) contains additional 173 amino acids at its N-terminus (the N-173 region). PTEN-long is a product of alternative translation initiation at a non-canonical start site (CTG), which is 519 bps upstream of the canonical ATG (Hopkins et al., 2013). The N-173 region is similar to the viral cell-penetrating protein Tat and allows PTEN-long to be secreted from and taken up by cells at distant locations in the body (Hopkins et al., 2013). A poly-alanine stretch within the N-173 region is essential to its secretion, while a poly-arginine stretch is critical for uptake (Hopkins et al., 2013). This N-173 region is largely disordered (Malaney et al., 2013b).

In CRCs, the AS takes place that affects a c-myc transcriptional suppressor, the far-upstream element-binding protein-interacting repressor [FIR, also known as Poly(U)-binding-splicing factor PUF60], that lack the transcriptional repression domain within exon 2 (FIRΔexon2) in CRCs (Rahmutulla et al., 2013). FIR is predicted to possess two long IDPRs (residues 1–126 and 300–500), with the N-terminal disordered domain being missing in AS isoforms. The high prevalence of AS in transforming growth factor beta receptor 2 (TGFBR2) has been recently reported in oral squamous cell carcinoma (OSCC) and in potentially malignant oral disorders (PMDs), with almost no such event detected in normal tissues (Sivadas et al., 2014). The AS of TGFBR2 occurred at the non-canonical splice sites and resulted in a selective exclusion of TGFBR2 mRNA sequences that code for TGFBR2 regions involved in tumor suppressive interactions such as ATM, CLK2, and CDC2, whereas the TGFBR2 sites related to the tumor promoting interactions such as interactions with MAPK1, PDK1, and PRKCZ were maintained in these aberrant transcripts (Sivadas et al., 2014).

As far as potential molecular mechanisms of the aberrant AS in cancer are concerned, recent study of human primary and metastatic colon cancer samples clearly indicated that the cancer proliferation can be driven by the overexpression or amplification of a component of the tri-small ribonucleoprotein (tri-snRNP) spliceosome complex, the pre-mRNA processing factor 6 (PRPF6), which commonly occurs in colon tumorigenesis (Adler et al., 2014; Lokody, 2014). Another recent study indicated that the switch of AS that occurs during epithelial-mesenchymal transition and is correlated with the aggressiveness of breast cancer correlate and breast cancer metastasis is promoted by the heterogeneous nuclear ribonucleoprotein M (hnRNPM), which is an RNA-binding protein that competes with an epithelial splicing regulator ESRP1 for binding to the same cis-regulatory RNA elements (Xu et al., 2014). Also, the activity of the spliceosome (a large macromolecular complex that mediates splicing and AS events) and accessory splicing factors is modulated by their reversible phosphorylation. The kinases and phosphatases involved in these PTMs significantly contribute to AS regulation, to its integration in the complex regulative network that controls gene expression in eukaryotic cells and its aberrations that characterize neoplastic transformation (Naro and Sette, 2013).

Finally, a case of extensive AS of the *TMPRSS2*-*ERG* gene fusion represents an important illustration of the combined effects of chromosomal translocations and AS (Hu et al., 2008; Sette, 2013). Here, a member of the ETS transcription factor family, ERG that is typically expressed at very low levels in benign prostate epithelial cells is fused with the androgen-responsive TMPRSS2 (transmembrane protease serine 2) to generate a prostate cancer oncogene. The resulting TMPRSS2-ERG hybrid causes abnormally high expression levels of the transcription factor in neoplastic cells. Furthermore, this fusion-derived gene was shown to undergo AS and generated multiple mRNA variants encoding both full-length ERG proteins and isoforms lacking the ETS domain. Notably, an increase in the abundance of transcripts encoding full-length ERG was shown to correlate with less favorable outcomes in prostate cancer patients (Hu et al., 2008).

***Aberrant alternative splicing in neurodegeneration***. Although numerous studies confirmed the existence of specific differences in AS profiles between normal and cancer tissues (Omenn et al., 2010, 2014; Shkreta et al., 2013), cancers are not the only set of diseases affected/promoted/caused by altered AS. In fact, brain has the greatest amount of AS of all human tissues, and therefore it is quite clear that the aberrant AS of brain proteins is associated with numerous neurodegenerative diseases. For example, in an autosomal dominant neurodegenerative disease called frontotemporal dementia and parkinsonism linked to chromosome 17 (FTDP-17), patients possess a twofold increase in the 4R:3R ratio of tau isoforms [i.e., isoforms containing four (4R) or three (3R) microtubule binding domains, respectively] leading to enhanced aggregation causing the disease (Philips and Cooper, 2000). In spinal muscular atrophy (SMA), constitutive AS of the survival of motor neuron gene (*SMN*) generates the SMNΔ7 isoform lacking the region encoded by exon 7. This SMNΔ7 isoform displays decreased self-oligomerization and is unable to participate in the assembly of small nuclear ribonucleic particles (snRNPs), thereby affecting the biogenesis and localization of spliceosomal snRNPs in the cell and dramatically reducing the ability of cells to produce functional mRNAs (Philips and Cooper, 2000). In Parkinson's disease, six genes, including *PARK2, SNCAIP, LRRK2, SNCA, SRRM2*, and *MAPT*, are affected by aberrant AS (Fu et al., 2013). One of these genes, *SNCA*, encodes the most studied neurodegeneration-related IDPs, α-synuclein, aggregation propensity of which is modulated by AS (Beyer, 2006; Beyer and Ariza, 2013). Furthermore, a link between the α-synuclein AS and risk of sporadic Parkinson's disease has been established (Pihlstrom and Toft, 2011). Regulated AS of another classical neurodegeneration-related IDP, tau protein, produces multiple isoforms controlling function of this protein in normal brain by influencing tau localization, conformation, post-translational modifications, availability, and affinity for microtubules and other ligands, whereas aberrations in tau splicing directly cause several neurodegenerative diseases, sporadic, and familial “tauopathies,” exemplified by AD, frontotemporal dementia with Parkinsonism (FTDP-17), Down syndrome (DS; trisomy 21), and myotonic dystrophy type 1 (DM1) (Andreadis, 2012). In general, the global transcriptome analysis by exon arrays and next-generation sequencing (NGS) techniques (e.g., RNA-Seq) revealed that many of the AS events are associated with neurodegenerative brain diseases (NBDs), such as AD and Parkinson's disease (PD) (Mills and Janitz, 2012).

In acetyltransferase p300 (which is a new component of cellular aggregates in α-synuclein positive Lewy bodies of patients affected by PD), a highly disordered regions was found that displays similarities with prion-like domains, is encoded as an alternative spliced variant independently of the acetyltransferase domain, and provides an interaction interface for various misfolded proteins, promoting their aggregation (Kirilyuk et al., 2012). AS of acetylcholinesterase (AChE) produces two neuronal tissue-specific splice variants, the synaptic or tailed form (AChE-T) with the C-terminal extension of the common core by a peptide containing a cysteine, which favors dimerization, and the read-through form (AChE-R), whose C-terminal extension over the common core is shorter than that of AChE-T and lacks cysteine (Zimmermann, 2013). AChE-T and its C-terminal peptides T14 and T30 were suggested to play a pivotal, non-hydrolytic role in neurodegeneration (Greenfield et al., 2008; Halliday and Greenfield, 2012).

The high degree of AS in the brain is associated with expression of a larger number of splicing regulators than most other tissues (de la Grange et al., 2010). Among these brain-specific splicing regulators are the members of the CUG-BP, Elav-like family (CELF), also known as Bruno-like (Brunol) proteins. Six CELF proteins (CELF1-6) and their AS isoforms are responsible for the regulation of AS of transcripts involved in neuronal function, and the dysregulation of CELF-mediated AS in the brain has been implicated in the pathogenesis of some neurological disorders (Ladd, 2013).

***Aberrant alternative splicing in cardiovascular diseases***. Multiple CVDs, such as cardiomyopathies, arrhythmias, and a number of inherited heart-related conditions are developed due to the altered AS of sarcomeric genes, ion channels, and cell signaling proteins (Lara-Pezzi et al., 2013). For example, clusterin (CLU) or apolipoprotein J is a chaperone for misfolded proteins that might contribute to survival by reducing oxidative stress. CLU maintains partially unfolded proteins in a state appropriate for subsequent refolding by other chaperones, such as HSPA8/HSC70. Several AS isoforms of CLU are encoded from a single gene located on chromosome 8 in humans. These isoforms are ubiquitously expressed in the tissues, and have been implicated in aging, neurodegenerative disorders, cancer progression, and metabolic/ CVDs including dyslipidemia, diabetes, atherosclerosis, and myocardial infarction (MI) (Park et al., 2014). CLU is predicted to have several long IDPRs (residues 1–110, 280–350, and 400–449). The highly disordered N-terminal domain is the CLU region most affected by AS. AS of the *IL1RL1* gene encoding interleukin-1 receptor-like 1 or ST2 protein produces two forms of the protein, a transmembrane (ST2L) form and a soluble form (sST2), the levels of which are elevated in serum of CVD patients with heart failure or MI (Willems et al., 2012). Aberrant AS of the L-type calcium channel (LTCC, a membrane ion channel most responsible for calcium entry and excitation-contraction coupling) is among genetic factors favoring Timothy syndrome, which is a malady characterized by multiorgan dysfunction including lethal arrhythmias, webbing of fingers and toes, congenital heart disease, immune deficiency, intermittent hypoglycemia, cognitive abnormalities, and autism (Splawski et al., 2004).

At the level of individual genes, the AS-produced isoforms were shown to play diverse roles during heart failure (Lara-Pezzi et al., 2012). Some of the illustrative examples include: the AS-driven generation of two non-functional variants of the sodium channel SCN5A (which, in the norm, controls cardioprotection by ischemic preconditioning) during heart failure (Shang et al., 2007); downregulation of the AS variant of the cell-cycle-regulated kinase (CCRK, which promotes cardiomyocyte growth and survival) in heart failure (Qiu et al., 2008); production of the AS variants of the troponin I gene that reduce contraction efficiency (Feng and Jin, 2010); and appearance of the AS-generated titin isoforms affecting the cardiac stiffness in individuals with dilated cardiomyopathy (DCM) (Makarenko et al., 2004); and the improvement of cardiac function after infarction by the CnAβ 1 AS variant of the phosphatase calcineurin (Felkin et al., 2011). Curiously, troponin I (Hoffman and Sykes, 2008), titin (Ma et al., 2006), and calcineurin (Dunker et al., 2001) are all known to possess functionally important IDPRs.

***Altered alternative splicing in diabetes***. Wolframin is an 890 amino-acid glycoprotein predominantly localized in the endoplasmic reticulum (ER) encoded by the *WFS1* gene. Various alterations in *WFS1* gene including mutations and AS are associated with autosomal recessive Wolfram syndrome, autosomal dominant low frequency sensorineural hearing impairment (LFSNHI) DFNA6/14, some psychiatric diseases, and diabetes mellitus (Cryns et al., 2003). In type 2 diabetes mellitus, the resistance to insulin is assumed to be caused by changes in the abundance of the AS isoforms of insulin receptor (Sesti et al., 2001). Normally, the insulin receptor exists in two isoforms, which are characterized by the absence (Ex11^−^) or presence (Ex11^+^) of a 12 amino acid sequence at the C-terminal tail of α-subunit caused by the AS of exon 11. The Ex11^−^ form binds insulin with two-fold higher affinity than the Ex11^+^, a form which is more abundantly expressed in target tissues from the type 2 diabetic patients. This alteration in the abundance of two forms might be related to the insulin resistance and the decreased sensitivity for metabolic actions of insulin in type 2 diabetes (Imai et al., 1997; Sesti, 2000; Sesti et al., 2001).

### Genetic factors: altered expression of IDPs and disease

As it was already pointed out, cells have evolved multiple complex mechanisms during transcription and translation to regulate the availability of IDPs (Gsponer et al., 2008). Since IDPs are important players in various signaling and regulatory networks, their tightly controlled availability represents a very important factor for the normal functioning of a healthy cell. It was also proposed that this tight control of the availability of IDPs might provide fidelity in signaling, regulation, and recognition by minimizing the likelihood of unwanted, non-functional interactions, and inappropriate sequestering of proteins into non-physiological protein complexes (Gsponer et al., 2008). In agreement with this hypothesis, a careful analysis of dosage-sensitive genes (i.e., genes which are harmful when over-expressed) revealed that the proteins encoded by these genes are often intrinsically disordered and that these genes are tightly regulated at both mRNA and protein levels, suggesting that this tight control prevents a potentially deleterious increase in protein concentration under physiological conditions (Vavouri et al., 2009; Babu et al., 2011).

#### Aberrant expression of IDPs and cancer

In solid tumors there is a markedly altered pH profile compared to normal tissues. One of the important players in corresponding pH regulation is the electroneutral Na^+^, HCO^−^_3_ cotransporter (SLC4A7, NBCn1), which is significantly over-expressed in human mammary carcinomas (Gorbatenko et al., 2014). Curiously, the N-terminal half of this 1214 residue-long protein is predicted to be mostly disordered. Altered levels of the collapsin response mediator proteins (CRMPs, C-terminal tails of which are predicted to be disordered), were reported for several malignant tumors, such as lung, breast, colorectal, prostate, pancreatic, and neuroendocrine lung cancer (Tan et al., 2014).

Recently, it was pointed out that several N-glycoproteins are differentially expressed in some cancerous diseases (Tuccillo et al., 2014). The illustrative examples of this phenomenon include up-regulation of alpha-1-antichymotrypsin in HCC (Ishihara et al., 2011) and non-small-cell lung carcinoma (NSCLC) (Zeng et al., 2010); down-regulation of insulin-like growth factor binding protein 3 in HCC (Chen et al., 2011) and NSCLC (Zeng et al., 2010); up-regulation of galectin-3-binding protein (Gal3BP or Mac-2 BP) in HCC (Chen et al., 2011) and ovarian cancer (Tian et al., 2011a); and increased expression of periostin in most ovarian cancer subtypes (Tian et al., 2011a) and aggressive prostate cancer (Tian et al., 2011b).

In cancers, high levels of the Sry-containing protein Sox2 are correlated with poor prognosis and increased proliferation of cancer stem cells (Liu et al., 2013). Sox2, a member of the family of high-mobility group transcription factors, is crucial for early development and maintenance of undifferentiated ESCs (embryonic stem cells). Together with other reprogramming transcription factors, Sox2 was predicted to be highly disordered (Xue et al., 2012c). Recently is has been shown that overexpression and gene amplification of Sox2 is associated with the development of squamous cell carcinoma in multiple tissues such as the lung and esophagus (Liu et al., 2013).

Kruppel-like factor 5 (KLF5) is a basic transcription factor binding to GC boxes at a number of gene promoters and regulating their transcription. This transcription factor has essential roles in cell cycle regulation, apoptosis, migration, and differentiation. Expression of KLF5 was found to be abnormal in many cancer types (Dong and Chen, 2009). As many other transcription factors (Liu et al., 2006), KLF5 is predicted to be mostly disordered.

In uterine leiomyoma, the most common tumors found in the women of the reproductive age which is characterized by the excessive extracellular matrix (ECM), levels of integrin-β 1 are significantly up-regulated (Chen et al., 2013). Second half integrin-β 1 is predicted to be mostly disordered and this prediction is supported by the structural analysis of this protein, since structure is known for residues 21–465 (e.g., PDB ID: 3VI3). The cellular levels of MDMX, which is a heterodimeric partner of MDM2 and a critical regulator of p53, are noticeably elevated in tumors with wild-type p53 (Li et al., 2012). Such elevated levels of MDMX contribute to p53 inactivation. Crystal structures are known for the N-terminal SWIB domain (residues 25–110, PMID: 2VYR) and the C-terminal region necessary for interaction with UBP2 (residues 429–490, PMID: 2VJE), whereas the reminder of MDMX is disordered.

Aberrant expression of various members of the S100 protein family (which is the largest subfamily of calcium binding proteins of EF-hand type containing at least 25 different members) is associated with pathogenesis of various cancers (Chen et al., 2014). Curiously, since these proteins display a unique pattern of tissue/cell type specific expression in the norm, different cancer types were shown to be characterized by opposite alterations in the levels of different members of this interesting family. For example, over-expression of S100A2, S100A3, S100A6, S100A8/A9, and S100A11 is related to several types of cancer, whereas other types of cancer are characterized by the under-expression of these same proteins (Chen et al., 2014). This remarkable variability in the outputs of the deregulation of levels of these proteins can be understood by taking into account their wide functional spectrum, which spreads from to proliferation, to apoptosis, to metastasis, to tumor microenvironment, and to cancer stem cells (Chen et al., 2014). Another important factor defining this variability is intrinsically disordered nature of S100 proteins (Permyakov et al., 2011). In fact, comprehensive bioinformatics analysis revealed that these proteins are enriched in intrinsic disorder, with 62% of them being predicted to be disordered by at least one of the predictors: 31% are recognized as “molten globules” and 15% are shown to be in extended disordered form (Permyakov et al., 2011).

Various cancers are associated with the altered expression of many subunits of the Mediator complex (Schiano et al., 2014). Mediator is an evolutionarily conserved large proteinaceous machine, needed for growth and survival of all cells. A high prevalence of IDPRs was reported for various subunits of Mediator from both *Saccharomyces cerevisiae* and *Homo sapiens*, especially in the Tail and the Middle modules (Toth-Petroczy et al., 2008). The IDPRs were shown to contribute to function of this important complex in at least three different ways, individually serving as target sites for multiple partners having distinctive structures, acting as malleable linkers between globular domains, and facilitating assembly and disassembly of complexes in response to regulatory signals (Fuxreiter et al., 2008; Toth-Petroczy et al., 2008). Over-expression of five out of 13 subunits of an important translation-related machinery, human translation initiation factor eIF3 (which interacts with the 40S ribosomal subunit, and promotes binding of tRNA^Met^_i_ and mRNA), is known to cause malignant transformation (Zhang et al., 2008; Le Quesne et al., 2010).

#### Altered expression levels of IDPs in neurodegenerative diseases

An illustrative example of the role of over-expression of a particular protein in the pathogenesis of a given neurodegenerative disease is given by the dual-specificity tyrosine phosphorylation-regulated kinase 1A (DYRK1A) in Down syndrome (Wegiel et al., 2011). In fact, DYRK1A over-expression deregulate multiple pathways in the developing and aging Down syndrome brain, affecting hundreds of proteins, including cytosolic, cytoskeletal, and nuclear proteins, transcription factors (Wegiel et al., 2011). Over-expressed DYRK1A directly leads to the hyperphosphorylation of tau and phosphorylation of AS factor controlling balance between the tau AS isoforms (Wegiel et al., 2011). Although DYRK1A is an enzyme and therefore is expected to be mostly ordered, computational analysis revealed that this important kinase contains two very long disordered tails decorating catalytic domain (residues 1–150 and 500–763). Alterations in expression of highly conserved filamentous proteins septins is associated with variety of neurological conditions, such as Alzheimer's and Parkinson's disease (Peterson and Petty, 2010). Dozens of different human septins are encoded by 14 loci (SEPT1-SEPT14), and all septin proteins contain highly conserved GTPase and polybasic domain regions and highly diverged N- and/or C-termini (Peterson and Petty, 2010), which are predicted to be highly disordered.

An altered expression of brain-derived neurotrophic factor (BDNF, N-terminal half of which is predicted to be disordered) was found in postmortem brains and serum from patients with schizophrenia, Alzheimer's disease and mood disorders (Carlino et al., 2013). Also, in addition to the abnormal hyperphosphorylation reported below (see “Aberrant PTMs and neurodegenerative diseases” section below), aberrant expression of the intrinsically disordered tau protein is known to be associated with AD and Down syndrome (Cardenas et al., 2012). The expression levels of glutamate receptor are dramatically altered in regions of the AD brain possessing the greatest pathological changes (Proctor et al., 2011). Another illustration of the role of protein overexpression in neurodegeneration is given by a classical pathology-related IDP, α-synuclein, where multiplications of the *SNCA* gene that encodes this IDP are associated with autosomal dominant PD (Eriksen et al., 2005; Elia et al., 2013). One of the causative agents of SMA, an autosomal recessive neurodegenerative disorder which is a leading genetic cause of infantile mortality, is deletion of a highly disordered protein, survival motor neuron-1 (SMN1) (Lorson et al., 2010).

#### Alterations of IDP expression in cardiovascular diseases

In CVDs, changes in expression levels of connexins (Cxs, which are intercellular channels forming low-resistance pathways permitting ions and metabolites up to 1 kDa in molecular mass to flow from cell to cell), such as Cx43, are known to modulate cell-cell coupling and the path of excitation spreading throughout the heart (Zhang and Shaw, 2013). C-tail of Cx43 (residues 230–382) is predicted to be highly disordered. Other proteins with altered expression and activity in major heart diseases, such as ischemic heart disease, cardiomyopathies, and congestive heart failure, are sarcoplasmic reticulum Ca^2+^ ATPases (SERCA) 2a and 2b, which are crucial for recycling cytosolic Ca^2+^ into the lumen of the sarcoplasmic reticulum (SR) (Shareef et al., 2014). Hart failure, which is the end stage of MI, cardiac hypertrophy, and hypertension, is characterized by the down-regulation of the alpha-myosin heavy chain (α-MHC) gene and SERCA genes and reactivation of specific fetal cardiac genes including atrial natriuretic factor and brain natriuretic peptide (Duygu et al., 2013). In addition to SERCA, altered levels and activities of many other Ca^2+^ cycling proteins are related to the heart failure. Among these Ca^2+^ cycling proteins responsible for the release and reuptake of intracellular Ca^2+^ that drives muscle contraction and relaxation are the ryanodine receptor 2, cardiac (RyR2), Ca^2+^ release channel macromolecular complexes, and phospholamban (Marks, 2013).

Altered expression of the angiotensin-converting enzyme 2 (ACE2) is associated with cardiac and vascular diseases, such as failing human hearts and atherosclerotic vessels. ACE2 degrades angiotensin II, which is the main effector of the classic renin-angiotensin system (RAS) that plays an important role in the pathophysiology of CVD (Burrell et al., 2013). Elevated levels of plasma plasminogen activator inhibitor-1 (PAI-1) have been shown to precede MI in patients (Juhan-Vague et al., 1996; Ploplis, 2011). In addition for being aberrantly expressed in various cancers, levels of the Kruppel-like factor 5 (KLF5) are altered in all vascular smooth muscle cell (VSMC)-related diseases, such as atherosclerosis, restenosis after angioplasty, cardiac hypertrophy, and hypertension (Dong and Chen, 2009).

**Figure 5:**
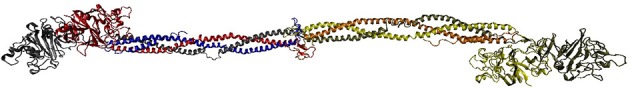
**Crystal structure of a hexameric complex formed by three fibrinogen chains, fibrinogen alpha (blue and orange chains), beta (red and yellow chains), and gamma (gray and tan chains) (PDB ID: 3GHG)**.

Alteration in the composition of ECM proteins, such as claudin-5, occludin, zona occludens proteins (ZO-1 and ZO-2), increased levels of some ECM proteins (such as osteopontin, chondroitin sulfate proteoglycan neurocan, and fibrinogen), and decreased levels of ECM receptors, such as integrins and dystroglycan, are associated with the altered blood-brain barrier (BBB) found in neurologic diseases, such as stroke (Baeten and Akassoglou, 2011). Osteopontin is an acidic hydrophilic glycophosphoprotein that functions as a cell attachment protein and was predicted to be mostly disordered and shown to be a typical IDP in solution by multidimensional nuclear magnetic resonance spectroscopy, synchrotron radiation circular dichroism spectroscopy, and small-angle X-ray scattering (Platzer et al., 2011). Many of the listed above proteins are predicted to possess long IDPRs. Massive parts of neurocan (residues 350–1050), fibrinogen alpha (residues 100–650), beta (residues 1–250), and gamma chains (flexible residues with disorder scores close to 0.6 are found in regions 65–177 and 318–349), occludin (residues 300–522), ZO-1 (residues 100–400 and 600–1600), ZO-2 (residues 100–500 and 900–1190), and dystroglycan (residues 300–500 and 800–895) are predicted to be highly disordered. In agreement with these predictions, no crystal structure is available for the monomeric forms of any fibrinogen chain, whereas crystal structure was solved for a hexameric complex formed by all three fibrinogen chains (PDB ID: 3GHG, Figure 5). Importantly, many residues were missing in this crystal structure (e.g., residues 1–26 and 201–562 in fibrinogen alpha chain, residues 1–57 and 458–461 in fibrinogen beta chain, and residues 1–13 and 395–411 in fibrinogen gamma chain) indicating that the corresponding regions remain high flexibility even in their bound forms.

#### Altered expression of IDPs in diabetes

Type 2 diabetes, a heterogeneous disorder with hyperglycaemia caused by impaired insulin secretion and decreased insulin sensitivity is characterized by aberrant expression of several important proteins in β-cells and pancreatic islets (Ostenson and Efendic, 2007). Among the islet proteins, whose levels are altered in type 2 diabetes, are GLUT2, glucokinase, phosphofructokinase, glucose-6-phosphoisomerase, m-glycero-phosphate dehydrogenase, pyruvate kinase, KCNJ11/Kir.2, SUR1, PDX-1, Foxo-1, and IRS-2 (Ostenson and Efendic, 2007). Markedly decreased expression of a number of SNARE complex proteins [such as syntaxin-1A, SNAP-25, VAMP-2, nSec1 (MUNC18), synaptophysin, and synaptotagmin V] was also found in pancreatic islets of type 2 diabetic patients (Ostenson et al., 2006). It has been established that the non-bound SNAP-25 is mostly disordered in solution (Fasshauer et al., 1997). In agreement with these data, recent comprehensive bioinformatics analysis revealed that human SNARE proteins possess substantial amount of predicted disorder (Pietrosemoli et al., 2013).

One of the hallmarks of the type 2 diabetes is diabetic dyslipidemia associated with the increased production of very low density lipoprotein (VLDL) (Verges, 2010). This abnormality is caused by the aberrant levels of hepatic apolipoprotein B (ApoB), a protein playing crucial role in the VLDL production by the liver (Verges, 2010). ApoB is a very large (4536 residues), mostly ordered protein that is predicted to have several relatively long IDPRs (e.g., residues 299–340 and 506–546). Skeletal muscle of type 2 diabetic patients shows reduced expression of mitochondrial proteins peroxisome proliferator-activated receptor gamma coactivator 1-α (PGC-1α), peroxisome proliferator-activated receptor gamma coactivator 1-β (PGC-1β), and the mitochondrial fusion protein mitofusin 2 (Mfn2) (Zorzano et al., 2010). Both PGC-1α and PGC-1β are predicted to be mostly disordered, and Mfn2 is expected to be mostly ordered, this protein is predicted to have multiple short IDPRs. Abnormal expression of insulin-like growth factor (IGF) binding proteins (IGFBPs) was detected in diabetes and could be used as a sensitive marker of insulin resistance defined as decreased sensitivity and/or responsiveness to metabolic actions of insulin promoting glucose disposal (Ruan and Lai, 2010). Several different IGFBPs are found in the serum, other biological fluids, and tissue extracts. The members of this superfamily of homologous proteins form an important link between the insulin and IGF systems (Ruan and Lai, 2010). Many members of this IGFBP superfamily are predicted to be either mostly disordered or at least possess long IDPRs.

One of the serious complications of diabetes is diabetic nephropathy (Molitch et al., 2003). In fact, 20–30% of patients with type 1 and or type diabetes mellitus possess nephropathy, which develops in 40–50% of patients with a 20-year history of type 1 diabetes mellitus (Thrailkill et al., 2009). One of the hallmarks of diabetic nephropathy is altered expression or activation of matrix metalloproteinases (MMPs), such as collagenases, gelatinases, stromelysins, matrilysins, and membrane-type MMPs, which have the capacity to breakdown all components of the ECM (Thrailkill et al., 2009). Another protein associated with diabetic nephropathy is angiotensin I-converting enzyme (ACE), elevated levels of which are found in this pathology (Lely et al., 2007). Although all MMPs and ACE are enzymes and are expected to be mostly ordered, they do possess regions of predicted disorder.

### Non-genetic factors promoting pathogenicity of IDPs

#### Abnormal posttranslational modifications

***Abnormal PTMs and cancer***. Functions of many IDPs and IDPRs are controlled, modulated, and regulated by various PTMs. Therefore, aberrant PTMs are commonly associated with several human diseases. In fact, all major PTMs, such as glycosylation, phosphorylation, acetylation, ubiquitination, methylation, and palmitylation, have been observed to be altered in cancer, affecting key cellular pathways including signal transduction, cell membrane receptor function, and protein-protein interactions (Markiv et al., 2012). For example, abnormal glycosylation of some glycoproteins due to deregulated glycosyltransferases and glycosidases is known to be a common phenomenon of many malignancies, including CRC, where elevated levels of the cell-surface α2,6-linked sialic acids have been linked to metastatic spread and therapeutic resistance of this cancer (Park and Lee, 2013). The widespread and diverse PTMs of histones, important nuclear IDPs (Peng et al., 2012) that are crucial for regulated gene expression and for a variety of epigenetic mechanisms, are under very tight and complex spatial and temporal control (Campbell and Turner, 2013). This spatial and temporal regulation of histone modifications is distorted in malignancies on both a genome-wide and discrete gene loci levels (Campbell and Turner, 2013). For example, excessive aberrant acetylation and methylation of specific histone residues have been found in CRC (Gargalionis et al., 2012). Also, alterations of different PTMs at lysine residues (such as acetylation, methylation, ubiquitination, and sumoylation) of proteins involved in DNA repair is often associated with genomic instability, which is the major cause of different diseases, especially cancer (Chatterjee et al., 2012). It has been even proposed that the analysis of histone modifications in circulating nucleosomes can be used for the diagnosis and estimation of prognosis in the CRC patients (Gezer and Holdenrieder, 2014).

Aberrant glycosylation is a well-established event in oncogenesis and cancer progression (Tuccillo et al., 2014), and many biomarkers used for diagnosis, prognosis, and prediction of many cancers are N-linked glycosylated proteins (Drake et al., 2006; Tian and Zhang, 2013). For example, malignant transformation of cells is associated with aberrant glycosylation of mucins, abnormal branching of *N*-glycans, and increased presence of sialic acid on proteins and glycolipids (Hauselmann and Borsig, 2014).

In addition to translational regulation discussed above, cancer-related activities of Sox2 protein are controlled by various PTMs (Liu et al., 2013). In fact, many sites of Sox2 can be modified through phosphorylation, acetylation, ubiquitination, methylation, and SUMOylation, and based on which sites are modified, Sox2 displays different normal and pathological activities (Liu et al., 2013). Ubiquitination-deubiquitination of a master transcriptional repressor that acts as a tumor suppressor or oncogene in diverse types of cancers, the RE-1 silencing transcription factor (REST) or neuron-restrictive silencer factor (NRSF), controls levels of this protein in cancer, and abnormal upregulation of REST has been found in medulloblastoma, neuroblastoma, and glioblastoma (Huang and Bao, 2012). As many other transcription factors (Liu et al., 2006; Guo et al., 2012), REST/NRSF is predicted to be highly disordered.

It is important to remember that alterations in PTMs of many disease-related proteins are typically produced by alterations of modifying enzymes. For example, aberrant phosphorylation, acetylation, methylation, sumoylation, and ubiquitination of the AR found in prostate cancer is caused by alterations of enzymes that modify the AR (Gioeli and Paschal, 2012). Also, histone acetyltransferases (HATs) and histone deacetylases (HDACs) are two classes of enzymes regulating histone acetylation whose altered activity has been identified in several cancers (Di Gennaro et al., 2004).

***Aberrant PTMs and neurodegenerative diseases***. In Huntington's disease (HD), a genetic neurodegenerative disorder caused by CAG expansions in the gene encoding Huntingtin protein (Htt), alterations of several histone PTMs are found, including phosphorylation, acetylation, methylation, ubiquitination, and polyamination (Moumne et al., 2013). Various PTMs of Htt itself, such as phosphorylation, sumoylation, ubiquitination, acetylation, proteolytic cleavage, and palmitylation, are also significantly altered in HD, resulting in changes in clinical phenotypes (Ehrnhoefer et al., 2011). In AD, which is a neurodegenerative disorder characterized by the progressive cognitive decline and by accumulation of insoluble aggregates of two proteins in the brain, amyloid-β (Aβ) and the microtubule-associated protein tau, Aβ levels and tau aggregation are impacted by altered sumoylation (Lee et al., 2013). Aberrant phosphorylation of the microtubule-associated protein tau is known to be associated with AD pathology and pathogenesis of other tauopathies (Hernandez and Avila, 2007). In fact, in AD, tau is abnormally hyperphosphorylated to a stoichiometry of at least three-fold greater than normal tau. This hyperphosphorylation is believed to be a major driving force for pathological tau aggregation, leading to the formation of a histopathological hallmark of the disease, paired helical filaments assembled from neurofibrillary tangles. Abnormal hyperphosphorylation and concomitant aggregation of tau is also a characteristic feature of several other tauopathies (Wang et al., 2013). Abnormal hyperphosphorylation of a RNA/DNA binding protein TDP-43 (TAR DNA binding protein 43) was shown to accumulate in the cytoplasm of neuronal cells of patients affected by fronto temporal lobar degenerations (Buratti and Baralle, 2008). TDP-43, which is known to control both normal and pathological RNA splicing events, is predicted to have a long disordered C-tail (residues 250–414). In Huntington disease, mutations in huntingtin (which normally undergoes different PTMs, such as phosphorylation, SUMOylation, ubiquitination, acetylation, proteolytic cleavage, and palmitoylation) significantly alters PTMs leading to changes in the clinical phenotype (Ehrnhoefer et al., 2011).

Prion diseases are characterized by the conversion of a soluble form of the glycoprotein prion protein (PrP^C^) to the abnormal (infectious) scrapie form (PrP^Sc^) (Gains and LeBlanc, 2007). NMR analysis revealed that human PrP^C^ is a hybrid protein that contains a C-terminal globular domain extending from residues 125–228, and an N-terminal disordered tail (Zahn et al., 2000). Recent study revealed that the transmissible spongiform encephalopathy (TSE) strain characteristics are affected by the glycosylation status of PrP (Cancellotti et al., 2013). This conclusion is based on the comparative study of the infectious properties of the isolates of three TSE strains passaged through transgenic mice with PrP devoid of glycans at the first, second or both N-glycosylation sites. This analysis revealed that the strain-specific characteristics of the TSE strain changed when PrP^Sc^ was devoid of one or both glycans suggesting that the PTMs of PrP may play a role in altering the infectious properties of a TSE strain (Cancellotti et al., 2013).

***Altered PTMs in cardiovascular diseases***. Altered PTMs of the voltage-gated Na channel isoform 1.5 (NaV1.5) contribute to the aberrant functions of this important protein associated with acquired cardiac disorders, including arrhythmias and heart failure (Herren et al., 2013). NaV1.5 is the pore forming α-subunit of the voltage-gated cardiac Na channel, which is responsible for the initiation and propagation of cardiac action potentials. Abnormal PTMs affect localization and gating potential of this protein (Herren et al., 2013). Abnormal phosphorylation of myosin light chain and troponins I and T, and, potentially, altered oxidation and glycation of sarcomeric proteins represent very important mechanisms underlying the myofilament dysfunction in DCM (LeWinter, 2005). Earlier, troponin I was shown to possess functionally important disordered regions (Hoffman et al., 2006). Furthermore, subsequent comprehensive bioinformatics analysis revealed that different troponin I isoforms are characterized by different abundance and distribution of intrinsic disorder (Hoffman and Sykes, 2008). This isoform-specific disorder variability was suggested to have potential mechanistic significance being responsible for modulation of the extent to which conformational fluctuations in tropomyosin are communicated to the troponin complex (Hoffman and Sykes, 2008). Troponin T is predicted to be mostly disordered.

Since constitutive nitric oxide (NO) synthases (NOS) are among the crucial factors responsible for the maintenance of myocardial Ca^2+^ homeostasis, myocardial relaxation and distensibility, and protection from arrhythmia and abnormal stress stimuli, the aberrant regulation and dysfunction of these important proteins in hypertension, hemodynamic overload, and atrial fibrillation are known to lead to the production of superoxide instead of NO (Carnicer et al., 2013; Shibata et al., 2013). Deregulation of NOS1 and NOS3 phosphorylation and glutathionylation are potent contributors to vascular disease, myocardial ischemia, reperfusion injury, MI, cardiac hypertrophy, and failure (Carnicer et al., 2013). NOS1 and NOS3 are predicted to have long IDPRs.

***Abnormal PTMs in diabetes***. Maintenance of metabolic homeostasis is controlled by some nuclear receptors, e.g., by the subfamily of the peroxisome proliferator-activated receptor (PPARs), which consists of three members (PPAR-α, PPAR-γ, and PPAR-δ) encoded by different genes and is involved in a wide range of physiological processes affecting lipid homeostasis, inflammatory responses, adipogenesis, insulin sensitivity, reproduction, wound healing, and carcinogenesis (Escher and Wahli, 2000; Lazennec et al., 2000; Mandard et al., 2004) PPARs has similar structural topology, possessing an N-terminal ligand-independent transactivation domain (AF-1), a two zinc-finger DNA-binding domain, a hinge domain, and a C-terminal ligand-binding domain containing a ligand responsive activation domain (AF-2). Structural analysis of PPAR-γ revealed that this protein has several long regions of missing electron density (PDB ID: 3DZY), such as residues 1–211, 238–298, and 443–477 (Chandra et al., 2008; Fuxreiter et al., 2011). The N-tails of PPAR-α (residues 1–96) and PPAR-δ (residues 1–70) are predicted to be disordered. Curiously, obesity (which one of the major underlying cause of the metabolic syndrome) was shown to promote phosphorylation of PPAR-γ at Ser273 (pSer273), and this PTM is correlated with the dysregulation of a subset of PPAR-γ target genes (such as the insulin-sensitizing adipokines such as adiponectin and adipsin), many of which are dysregulated in obesity (Choi et al., 2010).

Glycation of fibrinogen is a very common PTM detected in diabetes (Hammer et al., 1989; Henschen-Edman, 2001). This is an important finding since even modest degrees of fibrinogen modification can alter the rate of assembly of fibrin monomers into a fibrin clot and the fiber structure and packing, thereby contributing to the increased atherothrombotic risk associated with hyperhomocysteinemia and diabetes (Hoffman, 2008). The peculiarities of disorder distribution in various chains of fibrinogen were already discussed (see section “Alterations of IDP expression in cardiovascular diseases”).

Although the modification of nuclear and cytoplasmic proteins at their serine and threonine residues by the post-translational attachment of *O*-linked N-acetylglucosamine (*O*-GlcNAc) is crucial for the regulation of various cellular processes, prolonged increases in *O*-GlcNAcylation and sustained increases in *O*-GlcNAc levels have been implicated in glucose toxicity and insulin resistance (McLarty et al., 2013). It has been also emphasized that there is a complex interplay between phosphorylation and *O*-GlcNAcylation, as a result of which proteins can be both *O*-GlcNAcylated and phosphorylated, since *O*-GlcNAc and phosphate moieties compete for the same protein residues, serine, and threonine (McLarty et al., 2013). Obviously, this contributes a new level of complexity for protein regulation by PTMs under conditions of globally elevated GlcNAcylation, since increased GlcNAcylation was shown to affect phosphate stoichiometry at most of the sites involved in active phosphorylation-dephosphorylation cycling (Wang et al., 2008). Among 711 phosphopeptides for which phosphorylation dynamics was analyzed, GlcNAcylation resulted in lower phosphorylation at 280 sites and caused increased phosphorylation at 148 sites (Wang et al., 2008). Three hundred and eighty one proteins affected by such dual PTMization possessed a wide spectrum of biological functions, such as chaperones, cytoskeleton regulatory proteins, metabolic enzymes, kinases, transcription factors, and RNA processing proteins (Wang et al., 2008). From the list of functions it is clear that many of these proteins are either IDPs or hybrid proteins possessing long functional IDPRs. In fact, protein involved in chaperon functions, RNA binding and processing, and regulation of cytoskeleton and transcription are all expected to be disordered based on the associative study correlating Swiss-Prot functional keywords and protein intrinsic disorder (Vucetic et al., 2007; Xie et al., 2007a,b), and on existing experimental data for some members of these functional classes.

#### Abnormal proteolytic degradation

It is known that proteolytic digestion is orders of magnitude faster in unstructured as compared to structured protein regions (Polverino de Laureto et al., 1995; Fontana et al., 1997, 1986, 2004; Iakoucheva et al., 2001; de Laureto et al., 2006). Therefore, it is extremely important for the protein cleavage process that the sites of cleavage would be located in regions that lack structure or possess high structural flexibility.

Among the multiple modifications affecting aggregation propensity of the intrinsically disordered soluble protein tau and thereby contributing to the neurofibrillary pathology in AD and other tauopathies is abnormal truncation (Kovacech and Novak, 2010). In fact, the progression of AD is at least partially linked to the existence of several site-specific tau cleavages generating a multitude of various truncated forms. It has been shown that tau truncation alone is sufficient to induce the complete cascade of neurofibrillary pathology suggesting that proteolytical abnormalities in the stressed neurons and production of aberrant tau cleavage products are important events in the AD pathogenesis (Kovacech and Novak, 2010).

High levels of the truncated form Gli3^R^ of a glioma-associated oncogene 3 (Gli3) are related to various human limb malformations (Ruiz i Altaba, 1999; Hui and Angers, 2011). Glioma-associated oncogene family members 1, 2, and 3 (Gli1, Gli2, and Gli3) are specific transcription factors for controlling the signal transduction of the Hedgehog (Hh) pathway, one of the key regulatory networks involved in animal development (Jiang and Hui, 2008). A peculiar feature of the Gli family members is that the regulated proteolytic processing can convert some Gli proteins from a full-length transcriptional activator form into a truncated repressor form (Hui and Angers, 2011). The appearance of the Gl3^R^ form represents a result of limited proteolysis of Gli3 by the proteasome (Hui and Angers, 2011). Being transcriptional activators possessing multiple zinc finger motifs, all three members of the Gli family are predicted to be extensively disordered.

Proteolytic processing of cell-surface proteoglycans (PGs) known as ectodomain shedding of syndecans is related to the facilitation of cancer and promotion of the cancer cell motility and invasion thereby increasing aggressiveness of various tumors (Theocharis et al., 2010). Among the PGs with aberrant proteolytic degradation patterns in cancers are versican, aggrecan, brevican, decorin, perlecan, glypicans, and syndecans (Theocharis et al., 2010). The majority of these proteins are characterized by moderate to high levels of predicted disorder. In the crystal structure of human glypican-1 (PDB ID: 4AD7), residues 24–28, 350–361, 406–412, and 476–529 are located within the regions with missing electron density. Similarly, abnormal proteolytic degradation of collagens [which are known to be enriched in intrinsic disorder (Peysselon et al., 2011)] contributes to the cancer pathogenesis via multiple routs via affecting the efficiency of cancer cell invasion, activation of integrins, cancer cell proliferation, angiogenesis (Egeblad et al., 2010).

#### Defective trafficking of IDPs and hybrid proteins

It has been pointed out that defects in trafficking of some proteins might represent an important mechanism in many diseases (Delisle et al., 2004). Many of these pathogenic proteins with defective trafficking are IDPs or hybrid proteins with long IDPRs. An illustrative example is given by the low-density lipoprotein receptor (LDLR), mutations in which are associated with familial hypercholesterolemia (Delisle et al., 2004). It was pointed out that ≈50% of LDLR mutations act to disrupt receptor protein trafficking from the ER to the Golgi complex (Hobbs et al., 1990; Goldstein and Brown, 2001). Structural and bioinformatics analysis revealed that LDLR possesses long IDPRs. For example, residues 4–275, 457–464, and 714–788 (PDB ID: 3M0C) comprise regions with missing electron density, which typically correspond to IDPRs (Le Gall et al., 2007).

In cystis fibrosis, multiple mutations in cystic fibrosis transmembrane conductance regulator (CFTR) cause defective protein trafficking, where the mutated CFTR is retained in the ER (Cheng et al., 1990). The most well-studied mutation affecting normal trafficking of CFTR is the deletion of the codon for a phenylalanine at position 508 (ΔF508) of CFTR protein and is found in 70% of affected patients (Cheng et al., 1990; Delisle et al., 2004). Region 708–831 of CFTR, which is the functional regulatory (R) domain phosphorylation of which domain initiates Cl^−^ channel activity of CFTR, was shown to be predominantly disordered in solution (Ostedgaard et al., 2000).

Mutations in connexin 32 (Cx32), which is an integral gap junction membrane protein forming channels for the transmission of electrical signals and diffusion of small ions and molecules between coupled cells, are known to be associated with the Charcot-Marie-Tooth disease (CMTX) (Bergoffen et al., 1993; Delisle et al., 2004). Some CMTX mutations found in this complex polygenic neuropathic disorder, that constitutes the most common form of inheritable disease in the peripheral nervous system, result in the Cx32 protein that is synthesized but not properly transported to the plasma membrane (Delisle et al., 2004). C-terminal tail of Cx32 protein (residues 221–283) is predicted to be extensively disordered.

In the norm, the vasopressin-regulated aquaporin-2 channel (AQP2) is found in the kidney at distinct sites along nephrons and collecting ducts (Nielsen et al., 2002). Several mutations in AQP2 are associated with impaired transport from the ER and development of the non-X-linked nephrogenic diabetes insipidus (NDI), a hereditary malady characterized by the inability to concentrate the urine resulting in excessive urine production and thirst (Tamarappoo and Verkman, 1998) Being a transmembrane protein, AQP2 is expected to be mostly structured. However, computational analysis revealed that it has two long predicted IDPRs, residues 147–164 and 238–271.

## Concluding remarks

Intrinsic disorder is very common in proteins, plays various roles in numerous protein functions and is tightly controlled in the healthy cells. Among the major functions of IDPs are recognition as well as regulation and control of various signaling events. Normally, function and abundance of IDPs are tightly controlled. The breaking these proteins out of control brings havoc and initate various pathological events. In fact, IDPs and hybrid proteins containing ordered domains and functional IDPRs are commonly involved in the pathogenesis of human diseases. The crucial roles of IDPs and hybrid proteins in promoting and supporting the disease states are obvious from several illustrative examples of well-characterized disease-related IDPs, such as p53, α-synuclein, PTEN, tau protein, prion protein, etc., as well as from the results of comprehensive bioinformatics studies. The high degree of involvement of IDPs in the pathogenesis of various diseases is determined by the unique structural and functional properties of these proteins. IDPs and IDPRs frequently serve as major cellular regulators, recognizers and signal transducers. Their normal functionality is tightly controlled and modulated via a wide spectrum of PTMs and AS. Many IDPs/IDPRs can fold (completely or partially) upon interaction with corresponding binding partners and possess multiple binding specificities, enabling them to participate in one-to-many and many-to-one interactions.

Distortion of any of the mechanisms controlling IDP/IDPR functionality can be detrimental. Some disease-related proteins have an intrinsic propensity to form pathologic conformations, whereas other proteins require some external factors, such as impaired interactions with chaperones, intracellular or extracellular matrices, other proteins, small molecules, and additional endogenous factors, to gain conformational alterations leading to increased propensities for misfolding and dysfunction. Often, protein pathogenicity originates from altered splicing, chromosomal translocations, abnormal expression levels, or impaired trafficking. Formation of pathologic conformations can also be triggered by aberrant PTMs and increased degradation propensities. In other words, any cellular event that affects the functionality, foldability, abundance, or cellular distribution of these important players may cause pathological transformations.

### Conflict of interest statement

The author declares that the research was conducted in the absence of any commercial or financial relationships that could be construed as a potential conflict of interest.

## Abbreviations

Aβ: amyloid-β
ACE: angiotensin I-converting enzyme
ACE2: angiotensin-converting enzyme 2
AChE: acetylcholinesterase
AD: Alzheimer's disease
ALCL: anaplastic large cell lymphoma
α-MHC: α-myosin heavy chain
ALK: anaplastic lymphoma kinase
ALL: acute lymphoblastic leukemia
AML: acute myelogenous leukemia
APC: Adenomatous polyposis coli protein
ApoB: apolipoprotein B
AQP2: aquaporin-2 channel
AR: androgen receptor
AS: alternative splicing
BBB: blood-brain barrier
BDNF: brain-derived neurotrophic factor
BRCA1: breast cancer type 1 susceptibility protein
Brunol: Bruno-like
CARS: cysteinyl-tRNA synthetase
CELF: CUG-BP, Elav-like family
CCRK: cell-cycle-regulated kinase
CFTR: cystic fibrosis transmembrane conductance regulator
CIMF: chronic idiopathic myelofibrosis
CLTC: clathrin heavy chain
CLU: clusterin
CML: chronic myelogenous leukemia
CMML: chronic myelomonocytic leukemia
CMTX: Charcot-Marie-Tooth disease
CRC: colorectal cancer
CRMP: collapsin response mediator protein
CTA: cancer/testis antigens
CVD: cardiovascular disease
Cx: connexin
DCM: dilated cardiomyopathy
DCTN1: dynactin
DM1: myotonic dystrophy type 1
DS: Down syndrome (trisomy 21)
DYRK1A: dual-specificity tyrosine phosphorylation-regulated kinase 1A
ECM: extracellular matrix
ER: endoplasmic reticulum
ETV6: ETS translocation variant 6 (also known as TEL)
EWS: Ewing's sarcoma
FGFR1: fibroblast growth factor receptor 1
FGFR1OP: fibroblast growth factor receptor 1 oncogene partner
FIR: FUSE-binding protein-interacting repressor (also known as PUF60)
FTDP-17: frontotemporal dementia with Parkinsonism
Gli: Glioma-associated oncogene
HAT: histone acetyltransferase
HCC: hepatocellular carcinoma
HD: Huntington's disease
HDAC: histone deacetylase
Hh: Hedgehog
HLH: helix-loop-helix
hnRNPM: heterogeneous nuclear ribonucleoprotein M
Htt: Huntingtin protein
IDP: intrinsically disordered protein
IDPR: intrinsically disordered protein region
IGF: insulin-like growth factor
IGFBPs: insulin-like growth factor binding protein
IL1RL1: interleukin-1 receptor-like 1 (also known as ST2 protein)
KLF5: Kruppel-like factor 5
JAK2: Janus tyrosine kinase 2
KIF5B: kinesin 1 heavy chain
KLC1: kinesin light chain 1
KLF6: Kruppel-like Zn-finger transcription factor 6
MSN: moesin
LDLR: low-density lipoprotein receptor
LFSNHI: low frequency sensorineural hearing impairment
LTCC: L-type calcium channel
MDS: myelodysplastic syndromes
Mfn2: mitochondrial fusion protein mitofusin 2
MI: myocardial infarction
MMP: metalloproteinase
MYH9: myosin II heavy chain type A
NaV1.5: voltage-gated Na channel isoform 1.5
NBD: neurodegenerative brain disease
NDI: nephrogenic diabetes insipidus
NGS: next-generation sequencing
NO: nitric oxide
NOS: nitric oxide synthase
NPM: nucleolar phosphoprotein nucleophosmin
NRSF: neuron-restrictive silencer factor
NSCLC: non-small-cell lung carcinoma
*O*-GlcNAc: *O*-linked N-acetylglucosamine
OSCC: oral squamous cell carcinoma
PAI-1: plasma plasminogen activator inhibitor-1
PD: Parkinson's disease
PDGFRB: platelet-derived growth factor receptor beta
PG: proteoglycan
PGC-1α: peroxisome proliferator-activated receptor gamma coactivator 1-α
PGC-1β: peroxisome proliferator-activated receptor gamma coactivator 1-β
PJS: Peutz-Jeghers Syndrome
PMD: potentially malignant disorder
PMF: primary myelofibrosis
PPAR: peroxisome proliferator-activated receptor
PPFIBP1: protein tyrosine phosphatase receptor type F polypeptide-interacting protein-binding protein 1 (also known as liprin beta 1)
PrP: prion protein
PrP^C^: soluble form of PrP
PRPF6: pre-mRNA processing factor 6
PrP^Sc^: abnormal (infectious) scrapie form of PrP
PTEN: phosphatase and tensin homolog, deleted on chromosome TEN
PTM: posttranslational modification
PUF60: Poly(U)-binding-splicing factor (also known as FIR)
SCN5A: sodium channel 5A
SERCA: sarcoplasmic reticulum Ca^2+^ ATPase
SEPT: septin
SMA: spinal muscular atrophy
SMN1: survival motor neuron-1
snRNP: small nuclear ribonucleic particle
TEL: ETS translocation variant 6 (also known as ETV6)
TDP-43: TAR DNA binding protein 43
TFG: TRK-fused gene
TGFBR2: transforming growth factor beta receptor 2
TK: tyrosine kinase
TMPRSS2: transmembrane protease serine 2
TPM4: tropomyosin 4
tri-snRNP: tri-small ribonucleoprotein
RANBP2: RAN binding protein 2
RAS: renin-angiotensin system
REST: RE-1 silencing transcription factor
RET: REarranged during Transfection
RNF213: ring finger protein 213
RyR2: ryanodine receptor 2, cardiac
SQSTM1: sequestosome 1
TSE: spongiform encephalopathy
VCL: vinculin
VLDL: very low density lipoprotein
VSMC: vascular smooth muscle cell
ZO: zona occludens protein.
